# Adolescent and Young Adult (AYA) Cancer Survivorship Practices: An Overview

**DOI:** 10.3390/cancers13194847

**Published:** 2021-09-28

**Authors:** Silvie H. M. Janssen, Winette T. A. van der Graaf, Daniël J. van der Meer, Eveliene Manten-Horst, Olga Husson

**Affiliations:** 1 Department of Psychosocial Research and Epidemiology, Netherlands Cancer Institute, 1066 CX Amsterdam, The Netherlands; sh.janssen@nki.nl (S.H.M.J.); d.van.der.meer@nki.nl (D.J.v.d.M.); 2 Department of Medical Oncology, Netherlands Cancer Institute—Antoni van Leeuwenhoek, 1066 CX Amsterdam, The Netherlands; w.vd.graaf@nki.nl; 3 Department of Medical Oncology, Erasmus MC Cancer Institute, Erasmus University Medical Center, 3015 GD Rotterdam, The Netherlands; 4 Dutch AYA Care Network, 3511 DT Utrecht, The Netherlands; e.manten-ayanationaal@iknl.nl; 5 Department of Surgical Oncology, Erasmus MC Cancer Institute, Erasmus University Medical Center, 3015 GD Rotterdam, The Netherlands; 6 Division of Clinical Studies, Institute of Cancer Research, London SM2 5NG, UK

**Keywords:** adolescents and young adults, AYA, cancer, survivorship, AYA care programs

## Abstract

**Simple Summary:**

Adolescent and young adult (AYA) cancer patients, those who are diagnosed with cancer for the first time at the age of 15–39 years, are recognized as a distinct population within the oncology community due to the unique challenges they face throughout their disease trajectory. The number of AYAs who develop cancer per year has increased over the last decades, and >80% is expected to survive beyond 5 years. However, this rapidly growing AYA cancer survivor population is also at increased risk of cancer- and treatment-related long-term and late effects. There is a need for research efforts to inform the survivorship care of this unique population. The aims of this overview paper are to describe the epidemiology of the AYA cancer survivor population, the current knowledge on long-term and late effects, challenges and models of AYA survivorship care, and future opportunities within research as well as healthcare.

**Abstract:**

Worldwide, more than 1.2 million adolescents and young adults (AYAs; those aged 15–39 years) are diagnosed with cancer each year. Although considerable variability exists according to cancer site and stage of disease, the 5-year relative survival at the time of diagnosis has been estimated at >80% for all AYA patients with cancer combined. Extensive survivorship research in recent decades has focused on patients diagnosed with cancer as children (<15 years) and older adults (>39 years), yet few studies to date have reported outcomes specifically for patients diagnosed as AYAs. With increasing incidence and improving survival for many tumor types, leading to the majority of AYA patients with cancer becoming long-term survivors, there is a critical need for research efforts to inform the survivorship care of this growing population. This article describes the population of AYA cancer survivors according to their epidemiology and late and long-term effects, the challenges and models of AYA survivorship care, as well as future opportunities for research and healthcare.

## 1. Introduction

**Figure 1:**
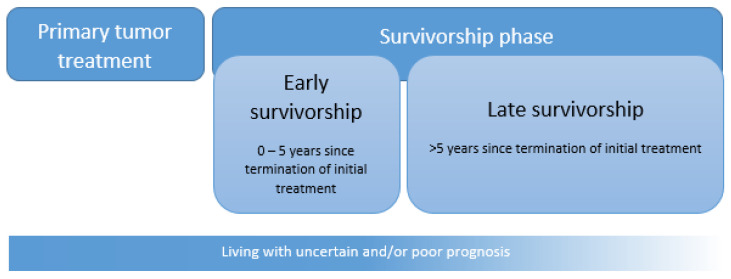
Primary tumor treatment, early survivorship, and late survivorship, partially adapted from the American Cancer Society [11]. Survivorship starts at the termination of the initial treatment. In the case of long-lasting adjuvant hormonal treatment after initial adjuvant chemotherapy, survival time, in our definition, starts at the end of chemotherapy. During survivorship, survivors attempt to transition to the ‘new normal life’, which can be subdivided into early and late survivorship [12]. Early survivorship covers the first 5 years since the termination of initial treatment [excluding maintenance therapy] and late survivorship from 5 years onwards since the end of initial treatment. For patients with a poor prognosis at diagnosis, survivorship starts at diagnosis. They have to live with a life-limiting disease.

Adolescent and young adult (AYA) [1,2,3] cancer patients, those who are diagnosed with cancer for the first time at the age of 15–39 years, are recognized as a distinct population within the oncology community due to the unique challenges they face throughout their disease trajectory: from symptom recognition, diagnosis and treatment to disease monitoring, advanced care planning, and survivorship [1,4,5]. Over the last decades, the incidence of cancer in AYAs has increased [6], and >80% is expected to survive beyond 5 years [7,8], meaning that there will be ever more AYA cancer survivors with the potential of many decades of life still to live. This rapidly growing AYA cancer survivor cohort is at increased risk of cancer- and treatment-related long-term and late effects [9]. There is, however, a paucity of evidence delineating these medical and psychosocial sequelae of AYA cancer survivors that are highly needed to underpin the design of AYA survivorship care programs and to ensure the quality of their survival. Currently, AYAs face several challenges in the transition from active cancer treatment to surveillance and early and late survivorship care [10]. The survivorship definition we use is depicted in Figure 1. The aims of this overview paper are to describe the epidemiology of the AYA cancer survivor population, the current knowledge on long-term and late effects, challenges and models of AYA survivorship care, and future opportunities within research as well as healthcare.

## 2. AYA Cancer Survivorship: Epidemiology

Approximately 1.2 million new cases of invasive cancer are diagnosed annually among AYAs [13], which is around 5% of all cancer diagnoses [7]. Over the last decades (2007–2016), cancer incidence increased slightly in all AYA age groups [14,15]. The distribution of different cancers varies substantially across the AYA age range. For example, pediatric malignancies, e.g., hematologic malignancies and brain tumors, are the most common cancers in adolescents (ages 15–19 years), while adult epithelial cancers, e.g., breast and colorectal cancers, are more common in older AYAs (ages 30–39 years), and there are also some cancers with the highest incidence among the AYA age group, e.g., germ cell tumors [7]. Triggered by the landmark report from the National Cancer Institute (NCI) in 2006, highlighting the dearth of improvements in survival rates for AYAs diagnosed with cancer compared with those diagnosed during childhood and adulthood—also called the “AYA gap”—the AYA group was defined as a high-priority group [1,16]. Fortunately, survival has improved more recently with a decline in overall cancer mortality during 2008 through 2017 by 1% annually [15]. The five- and ten-year relative survival (RS; defined as the ratio of observed survival in cancer survivors by the expected survival in a comparable general population matched on age and sex) of AYAs is now similar across age groups for all cancers combined [7,14,15]. Table 1 provides a brief overview of the age-standardized 5- and 10-year RS of AYA cancer survivors per cancer type, based on data from the American Surveillance, Epidemiology and End Results (SEER) 18 database of the NCI. Methods are described in Appendix A. Notable differences by tumor types and within some cancers are found in relative survival, reflecting differences in progress made, histologic subtype distribution, and available treatments. The distribution of tumor types among AYA cancer survivors has important consequences for the organization of AYA survivorship care, as risks for cancer recurrences, second primary malignancies, and treatment-related long-term and late effects will significantly impact supportive care needs, surveillance strategies, and follow-up care. In addition, despite the progress made for several cancers, there is also a group of patients living with an uncertain and/or poor cancer prognosis (UPCP). This includes patients undergoing established treatment with a life expectancy of 1 to 5 years (e.g., colorectal cancer stage IV), low-grade glioma survivors with a life expectancy of 5 to 10 years, and “new survivors” undergoing novel treatment(s) with uncertain prognosis (e.g., melanoma stage IV) [17]. Common challenges among AYAs with a UPCP include the loss of control of their life and disruption of developmental milestones, (unknown) effects of constant, aggressive, or novel treatments, a sense of social isolation (within the healthcare system and from peers), a constant balance between hope and risks, a lack of evidence-based prognostic information (e.g., due to a lack of focus on this specific patient group) and experiencing uncertainty about treatments (effectiveness and availability) [17]. These patients could potentially also benefit from AYA survivorship care programs.

## 3. What Sets AYA Cancer Survivors Apart?

AYAs with cancer are distinct from the pediatric (<15 years) and the older adult (>40 years) cancer populations, not only with respect to their spectrum of cancer types, but also the biology of their cancers, their developmental status, their particular psychosocial needs, and the long-term complications of their cancer and treatment thereof [1,7,16,18]. The following aspects may all impact survivorship outcomes and have implications for the follow-up care of AYAs with cancer:

- The percentage of AYAs with cancer who carry pathogenic variants in genes that predispose to cancer is significant [18], with important consequences for an individuals’ surveillance strategy.
- There is insufficient awareness of cancer risk and symptoms among AYAs and healthcare professionals resulting in prolonged and complex diagnostic trajectories, negatively impacting quantity and quality of survival [19,20].
- Despite similar histological diagnoses, tumor genomics and biology can differ between AYAs and older adults, leading to different medical treatment strategies [21,22].
- The biology of the host may differ according to age, with potential distinct pharmacokinetics and pharmacodynamics between children and AYAs/adults and potential impact on therapy efficacy and toxicity profiles [18,23,24,25].
- In contrast to the emphasis put on late effects of treatment in pediatric cancer patients, for AYAs hardly any late effect clinics are in place, and the lasting (physical) impact of treatments at this age is only known to a limited degree [26].
- Adolescence, emerging, and young adulthood are complex, unique phases of life due to the many physical, emotional, cognitive, and social transitions [27]. A cancer diagnosis during the AYA life stage exacerbates typical developmental challenges and interferes with the attainment of important age-specific milestones [16], including identity-forming, establishing autonomy, responsibility and independence, finishing education and starting a career, obtaining romantic relationships and starting a family [27]. In addition, the type of informal caregivers may differ among cancer patients with varying ages and life courses [28]. The way in which AYA cancer patients adjust to their cancer experience might have life-long implications for the quality of their survival time [29,30,31,32,33].

## 4. What Do We Know about Long-Term and Late Effects among AYA Cancer Survivors?

Much of what we have learned about AYAs to date has been extrapolated from research on childhood and older adult cancer patient populations in disease- but not age-specific cohorts, e.g., breast cancer and Hodgkin’s [16,34,35,36]. However, there is still a dearth of literature on (long-term) adverse health outcomes in AYAs treated for other early-onset adult cancers, such as colorectal cancer, and AYA cancers, such as melanoma [37,38,39,40,41]. We summarize the most important literature according to the biopsychosocial model, which provides a comprehensive framework for the physical, psychological, and social issues caused by cancer (treatment) and the interactions between different issues.

### 4.1. Physical Issues

Which (long-term) physical issues develop mainly depends on the location of the primary tumor and the type of treatment received [42,43,44]. Compared to siblings and the general population with no history of cancer, AYAs are at greater risk of potentially life-threatening chronic medical conditions [43,44,45]. These long-term complications can lead to significant medical expenses and healthcare utilization [45].

#### 4.1.1. Secondary Malignancies

AYA cancer survivors are at increased risk of developing secondary malignancies as a consequence of their first cancer diagnosis and its treatment [9,46,47,48,49]. For instance, survivors of Hodgkin’s lymphoma have, compared to the general population, a higher risk of developing secondary solid cancers due to treatments such as radiotherapy. This elevated risk can even persist for more than 25 years [50,51]. The observed increased risk of secondary cancers after Hodgkin’s lymphoma treatment has led to adaptations in treatment schedules, hence a reflection of the importance of survivorship research. Overall, well-known secondary malignancies for patients exposed to (high doses of) radiotherapy in certain areas or alkylating agents include, amongst others, breast, lung, stomach, and colorectal cancer, and leukemia. A better understanding of the exposure-related risk of other (newer) treatments, other concurrent risk factors, and appropriate screening measures are needed for secondary malignancies risk management among AYA cancer survivors [9,47,52].

#### 4.1.2. Cardiovascular Disease

Cardiovascular disease (CVD; including, amongst others, vascular disease, coronary artery disease, congestive heart failure, and cerebrovascular disease) can present during treatment as well as during late survivorship and contributes to increased risks of both morbidity and mortality [9,53]. Multiple risk factors are involved in the development of CVD, including treatment-related factors (e.g., high-dose anthracycline chemotherapy, heart within radiotherapy field), preexisting risks factors as well as secondary factors, such as one’s behavior [9,53]. Long-term survivors of testicular cancer who are treated with chemotherapy (e.g., cisplatin-containing) are at increased risk for cardiac events and may develop an unfavorable cardiovascular risk profile, including but not limited to hypertension and overweight [54,55]. The risk of developing CVD is twofold higher among AYA cancer survivors compared to their matched healthy controls and increases with older age [9].

#### 4.1.3. Endocrine Dysfunction

In AYAs, an increased risk of endocrine dysfunctions can occur due to changes in one’s metabolism or dysfunction of the gonads or thyroid, for example [9,56]. Thyroid dysfunction (e.g., hypo- and hyperthyroidism) is a common late effect for patients undergoing radiotherapy given the radio-sensitivity of the thyroid gland, of which the risk can persist even 20 years post-treatment [9]. Diabetes is another endocrine dysfunction, which frequently occurs as a treatment-related late effect and, besides thyroid dysfunction, is known as a leading cause for hospital visits [9,57]. Compared to the general population, AYAs have a 29% increased risk of developing diabetes. Besides lifestyle factors (e.g., poor nutrition, physical inactivity), treatments including abdominal radiotherapy and chemotherapy can contribute to an increased diabetes risk as well (e.g., insulin resistance, metabolic syndrome, changed pancreatic function) [9].

#### 4.1.4. Neurocognitive Deficits

Cancer (treatment) related neurocognitive deficits include problems regarding attention, memory, and executive functions [9]. As the brains of many AYAs are still in development, they are especially vulnerable to cognitive dysfunction [58]. These cognitive issues may eventually lead to problems in everyday life, including completing education or maintaining employment. Subsequently, this may impact psychological well-being. AYA cancer survivors have, compared to the general population, higher rates of cognitive dysfunction [59]. Especially AYAs with primary brain tumors or metastases treated with cranial radiotherapy and chemotherapy directed at the central nervous system should be considered at risk and screened for impaired neurocognitive outcomes [9].

#### 4.1.5. Fertility

Fortunately, many AYA cancer survivors were able to have children without the need for fertility preservation, and, over time, more attention has been paid to the potential impact of cancer (treatment) on fertility. However, cancer treatments (chemotherapy, surgery, or radiotherapy) still have the potential to destroy reproductive cells and can place both women and men at risk of fertility issues, depending on dosage and field, and age at exposure [9,36,41,46,60,61,62,63]. Issues include azoospermia among men and acute ovarian failure, premature ovarian insufficiency, and premature menopause among women [9,60,61]. Although some patients of younger ages may feel as if fertility is not relevant to them yet, it is important though to discuss the possibility of infertility, as it may have a huge impact later in life [16,64]. Unfortunately, despite the availability of fertility preservation options (e.g., embryo, oocyte, or ovarian tissue cryopreservation and hormonal therapies for women, or testicular tissue cryopreservation and sperm banking for men), the understanding of fertility risks may either not be complete or accurate, or the experience of discomfort among healthcare providers or patients may lead to the start of treatment without fertility preservation or fertility concerns among patients [16,31,46,65,66,67,68]. From the perspective of AYA survivors and their families, common barriers to fertility care include the lack of high-quality information regarding fertility risk and preservation, concerns regarding treatment delay, and concerns about the health of the AYA and their offspring. In addition, healthcare providers (HCPs) might make assumptions regarding the fertility interest of the patient that may be incongruent with the perspective of the AYA [68,69]. The use of a risk stratification model, including the risk for infertility in AYA cancer survivors and improving access to fertility preservation services, amongst others, may improve this process [9].

#### 4.1.6. Sexual Dysfunction

Erectile dysfunction, ejaculation issues, vaginal atrophy, and changes in sexual desire and orgasm sensitivity may all be consequences of, for example, changes to blood vessels or nerves in the genital area due to treatments such as surgery and radiation. Chemotherapy might alter hormone levels leading to early menopause (including loss of elasticity, shrinking, and vaginal dryness). Altogether, sexual dysfunction can result in intercourse not being comfortable or interference with one’s interest or joy in sex [70]. Besides these direct effects, there may also be indirect treatment effects (e.g., fatigue) or changes in one’s physical appearance or self-esteem that may negatively impact one’s sexual functioning and (their partner’s) sex life [31,71]. A study among Swedish AYAs, aged 15–29 and at least 1-year post-treatment, showed less satisfaction with sexual functioning among AYA cancer survivors compared to their healthy peers [72]. Although AYAs express a need for information regarding sexual health, this is not always recognized by HCPs [16,73]. They may feel discomfort or not confident in providing appropriate sexual healthcare. Furthermore, they may have misconceptions regarding AYAs’ sexual behaviors or think AYAs do not want to talk about it [16,73]. Even though AYAs report positive outcomes when discussing sexual health with their HCP, there are still no standard sexual health practices addressing AYAs’ needs yet [16].

#### 4.1.7. Body Disfigurement

Physical appearance is extremely important for AYAs, and related dissatisfaction is common [72,74]. AYAs are characterized as being very conscious of their body and appearance, which adds up to the vulnerability of this population. Especially young AYAs whose bodies are still in development may be more affected by treatments such as surgery or radiotherapy [41]. Disfigurement, the state of having one’s appearance deeply and persistently harmed medically due to cancer treatment, may include, among others, amputation (e.g., breast cancer or sarcoma survivors), scars, and chronic skin changes (e.g., head and neck cancer survivors), having an ostomy (e.g., colorectal cancer survivors) and having prosthesis [72,74,75,76]. Disfigurement negatively impacts one’s body image [72,74], which in turn may lead to feelings of insecurity and alienation from peers [31,71].

#### 4.1.8. Physical Condition

Treatments including radiation and chemotherapy and, indirectly, treatment-related effects as shortness of breath and fatigue may influence survivors’ level of physical activity and lead to a poor physical condition [77]. A Swiss study among AYAs observed significantly lower physical health compared to the general population [30], and Tai and colleagues showed significantly more AYA cancer survivors being obese [56]. Furthermore, in an American study, Ketterl and colleagues showed increased odds of physical impairment of work-related tasks among patients of several cancers, including sarcoma and central nervous system cancers, treated with surgery [78].

### 4.2. Psychological Issues

Most important psychological survivorship issues include psychological distress, posttraumatic stress and fear of cancer recurrence [41,46,59,66,77,79,80,81]. Psychological distress is known to be common and significantly greater among AYAs than older adults: AYAs understand the severity of the situation but may lack sufficiently developed distress tolerance, emotion regulation, and/or interpersonal effectiveness skills [41,77,82,83,84]. This stresses the importance of delivering developmentally appropriate support, as AYAs try to accomplish key developmental tasks but at the same time may have different cognitive and emotional developmental levels than older adults [41]. Compared with healthy matched controls and siblings, female AYA cancer survivors have a higher risk of developing depression and anxiety [43,77]. Results of the AYA HOPE study indicate that more than 50% of AYAs who were in need of mental health services did not receive these [85]. Kwak and colleagues reported increased psychological distress levels among AYAs compared to population norms at the time of diagnosis and after 12 months of follow-up while transitioning into survivorship [86]. Unfortunately, AYAs may delay the reporting of symptoms of distress to HCPs, which may be due to their coping style or fear of stigmatization [31]. In a Canadian study among AYA cancer survivors, help for emotional concerns was not sought because survivors either did not want to ask for it, felt embarrassed or thought nothing could be changed because someone had told them it was normal [80]. A significant minority (40%) of the Canadian AYAs struggled to cope with everyday life challenges. A lack of age specific services and interventions may hinder AYAs in trying to access support services [80].

Furthermore, research has shown that fear of recurrence is more prevalent among AYAs compared to older adult survivors [87,88]. AYAs want to learn how to handle and manage the fear of recurrence, which is shown to be an unmet information need [88]. In general, cancer patients with high fear of recurrence also experience significant psychological distress and have increased use of health services, while others change their behavior by, for example, avoidance or excessive self-examination [89]. Self-examination behaviors may be a signal for a survivorship care need as the AYA needs to know which signs and symptoms to track and when to contact an HCP. Compared with healthy matched controls and siblings, AYA cancer survivors also have a higher risk of developing posttraumatic stress [43,77]. In the end, the health-related quality of life (HRQoL) of AYA cancer survivors may be significantly impacted [33,90]. In an Australian survey, both AYAs and parents self-reported elevated levels of general distress and posttraumatic stress symptoms: rates of emotional distress were comparable between parents and AYAs [91]. This underscores the importance of being aware of the impact a cancer diagnosis may have besides solely on the patient, such as, for example, on caregivers.

Despite the negative psychological survivorship issues possibly experienced after cancer (treatment), there are also AYAs experiencing positive outcomes, such as resilience, post-traumatic growth, and appreciation for life. Although its definition varies, resilience is often defined as the ability to cope with the negative effects of a stressful experience such as cancer [29,92]. Factors that can either contribute to or inhibit resilience include stress and coping, goals, purpose and planning, optimism, gratitude and meaning, and connection and belonging [92]. The balance of these factors can be shifted in case of specific experiences or moods, for example, but when in balance, resilience may empower AYAs and lead to improved psychosocial outcomes [29,92]. Resilient AYAs are less likely to report unsatisfied counseling needs compared to those with less resilience [79]. Post-traumatic growth (PTG) is described as benefit finding—a change in view of life, feeling stronger or more confident. In other words, there is a positive psychological change as a result of one’s cancer experience [29,93]. PTG can be seen in relationships, perspective on life, or one’s self, or in spiritual beliefs [29]. Enduring stress is negatively associated with PTG, while social support is positively associated with PTG [29]. Both resilience and PTG are positively associated with satisfaction with life and HRQoL [29]. In qualitative studies, most AYAs report at least some resilience or PTG, although exact prevalence rates are lacking due to the heterogeneity of studies, definitions, applied theoretical frameworks and used tools [29].

### 4.3. Social Issues

A patient’s social role (fulfilling a recognized position in society, e.g., parent, employee, student) may alter due to cancer (treatment): participation in social activities may be limited and social maturation may be disrupted [32]. Due to the long-term effects of treatment, AYAs may find it difficult to make or maintain social contacts and an active and independent life [32,41,94]. Social functioning has been shown to be more challenging for AYAs compared to an age-matched population [32]. Missing out on life experiences such as going to college, dating, becoming independent, or having children can all lead to feelings of isolation and alienation, which is common among AYAs as it sets them apart from their peers while becoming more dependent on parents or caregivers [41,71,95]. AYA cancer survivors indeed perceive themselves as being different from their peers who had no cancer diagnosis [32,96], and as their healthy peers may not be familiar with cancer, this effect may be amplified [97].

#### 4.3.1. Education, Employment, and Financial Challenges

Although many AYAs continue or return to their education during or after cancer (treatment), as is the case for employment, educational trajectories may be disrupted due to being absent or unable to complete exams, amongst others [98,99], which may have a crucial impact on AYAs’ career development [100]. Although the impact of cancer (treatment) on education shows varying study results [56,101,102], AYAs indicate receiving inadequate educational support during their treatment and are in need of support with returning to school [31,103]. In a qualitative study among Danish AYAs, aged 15–25 at diagnosis, results indicate that AYAs experienced problems regarding their return to secondary or higher education due to the consequences of treatment, misunderstanding from their peers, and academic/municipal system barriers (e.g., complex navigation, miscommunication) [104]. Sources of support, such as parents, family members, and guidance counselors, were helpful in alleviating the burden of these problems [104].

In addition to educational challenges, returning to work after being absent due to treatment is also known to be a challenge for patients in general, while work addresses their financial needs, makes them feel productive, and suggests recovery and return to ‘normal life’ [73,97]. Compared to their peers, AYAs feel left behind in their employment trajectories [31]. Significantly more AYAs report being out of work or unable to work (24%) compared to their healthy peers (14%) [56]. In a study among Canadian AYA cancer survivors, respondents most often did not seek help for practical concerns such as returning to school or work because they did not want to ask for help or were either not aware of available services or where to go for these [80]. In some cases, AYAs experience discrimination from employers [31].

Interruptions in school or work may lead to significantly more distress [105] and eventually, in the long term, influence career opportunities and financial status [41,46], while this latter can already be impacted earlier on due to insurances and healthcare expenses [95]. Adding to this, AYA cancer survivors are known to face higher medical expenditures compared to their healthy peers [46,56,73] and are more likely to face financial problems than older cancer patients [101,106]. These problems may lead to loss of control and more (financial) reliance on others, including partners or parents [31,66,97,98]. Some AYAs may also avoid routine medical care due to these financial barriers [46,107]. However, given the nature of heath care provided (governmental primarily in Europe and Canada; private insurance and Medicaid in US), much of the access to care and financial toxicities will differ between continents and countries, although there will also be overlapping issues.

Important to note is that a change in education or employment status can be perceived both negatively and positively as some AYAs may deliberately chose for the change as their perspective on life has changed due to the cancer experience [108,109]. Future plans are re-evaluated due to the focused attention one has regarding purpose and reprioritized goals [99,109]. Vetsch and colleagues report that AYAs’ education or vocation can be of equal or even greater importance to them after their diagnosis than before. Some vocational goals of AYAs are directly informed by the experience of their own cancer diagnosis [109].

#### 4.3.2. Relationships

A cancer diagnosis affects more people than only the patient him/herself, which can either create a distance or enhance a relationship [110]. AYAs report a need for information regarding communication with others if they felt cancer had impacted their close relationships [111]. Kirchhoff and colleagues reported fewer AYAs being married, and young adult cancer survivors were more likely to divorce or separate from their partner than those without a cancer history (a 77% higher risk of divorce or separation) [112]. For those who are married or in a relationship, a cancer diagnosis may not only affect the relationship in its entirety, the partner may also experience a change in role functioning and future plans may be disrupted, and feelings of distress may arise. Within a study among breast cancer survivors aged 40 years or younger, the partnership was suggested to be an important source of support, which was associated with greater HRQoL levels [113]. In addition, starting a new relationship with a partner was considered challenging, due to feeling ‘abnormal’, fertility issues, low self-esteem, or a negative body image [110,114,115].

While there may be a negative impact on relationships due to the cancer experience, some AYAs also report a positive change, including more meaningful interpersonal relationships [93,99]. The whole cancer experience was beneficial for the relationship as it strengthened the relationship. A study by Bellizzi and colleagues showed that most AYAs experienced a positive impact on relationships with their spouses, parents, and siblings [99].

Altogether, the findings of these studies highlight the urgent need to better understand, screen for, and risk stratify, prevent and/or treat the negative long-term and late effects [16,41,43,64,73,116,117]. Many cancer-related sequelae experienced by AYAs have complex etiologies involving multiple overlapping mechanisms, making them difficult to prevent and treat [16,66,118]. This complexity, however, together with AYAs’ overall long-term survival [119], creates a strong rationale for exploring multidisciplinary (e.g., sexologist, occupational physician, (neuro) psychologist) AYA survivorship care, which includes prevention and treatment strategies for late effects.

## 5. Challenges and Models of AYA Survivorship Care

In many parts of the world, AYAs with cancer faced disparities of care as they were poorly served (not patient-centric) by the traditional dichotomy of pediatric and adult oncology services [90]. Fortunately, this gap in unmet care needs and poor outcomes of AYAs has been recognized, which led to the development of a new medical discipline called ‘AYA oncology’ [26] and the start of dedicated AYA care programs in some countries [12,25,73,120,121,122,123,124,125]. Overall, several essential components of AYA care programs are described in the literature, including (1) patient focus, (2) multidisciplinary approach, (3) cooperating pediatric and adult expertise, (4) staff education and training, (5) dedicated AYA space, (6) patient and family advocacy, and (7) research and clinical trial availability [12,25,123,126,127,128,129]. Although countries handle different age ranges for their AYA programs (varying from 15–25 years in Australia to 15–39 years in the United States and 18–39 years in the Netherlands), which may have consequences for the type of care provided, the patient focus remains an essential part in AYA care [12,123]. The multidisciplinary team (MDT) approach, including clinicians, clinical nurse specialists, and psychosocial/allied healthcare professionals, benefits AYAs due to the complexity and variety of care (needs) [125]. As referral pathways and services may not be directly available or visible to AYAs, who are often inexperienced in navigating the healthcare system, the help of healthcare teams are of great importance to guide AYAs through the system. Facilitating the cooperation between pediatric and adult medical oncologists for relevant tumor types is of importance as the best treatment regimen (pediatric or adult) needs to be chosen [12,123,125,127,130,131,132]. The benefits of such collaborative effort have been demonstrated for young AYA acute lymphoblastic leukemia patients, which showed superior survival outcomes when treated in a pediatric cancer setting compared to an adult cancer setting [133]. In addition, educating staff is of importance as HCP may lack AYA specific experience, which may affect the provision of care to this unique population [12,123,128]. Up till now, there is no optimal survivorship care model, and many differences (between and within countries) may exist, leaving much room for improvement and still a lot can be learned from each other (e.g., by benchmarking). Despite the emergence of AYA care programs, there is a limited number of programs with survivorship elements [1,25,73,77,134,135,136,137].

### 5.1. How Is Survivorship Care Organized for Children and Older Adults?

#### 5.1.1. Survivorship Care for Children

Pediatric cancer care tends to be family-centered, in which the child, parents, and physician are involved in the decision-making process [25]. Holistic, multidisciplinary care for pediatric cancer patients is often provided centralized in pediatric cancer centers, where both treatment and follow-up care are provided [25]. In general, pediatric cancer care is relatively well resourced, with overall a large amount of time available per patient and with a high staff/patient ratio [126]. As most pediatric cancer patients are treated intensively, they are at risk for early and/or late adverse medical and psychosocial effects. The Childhood Cancer Survivor Study showed that 62% of childhood cancer survivors had any mild/moderate chronic condition 5 to 14 years after diagnosis, which increased to 81% at 25 to 36 years after diagnosis. In addition, 27% of childhood cancer survivors had any severe/life-threatening/disabling chronic condition 5 to 14 years after diagnosis, which increased to 42% at 25 to 36 years after diagnosis [138]. Most pressing problems include treatment-related second cancers, endocrine disorders (hypothalamic-pituitary dysfunction, primary thyroid dysfunction, metabolic syndrome, decreased bone mineral density) [139], and other coexisting medical conditions typically depending on treatment-specific factors [34,140,141,142]. The large body of knowledge about cancer (treatment) related effects among childhood cancer survivors has led to exposure-related and risk-based long-term follow-up guidelines specific for this group of survivors, made available by the Children’s Oncology Group [143,144]. Different models of survivorship care are available for providing long-term follow-up care for childhood cancer survivors: cancer center-delivered care (high-risk survivors), shared care between treatment hospital and local hospital or primary care, and primary care physician-led/community-based follow-up (low risk survivors) [145].

#### 5.1.2. Survivorship Care for Older Adults (>40 Years)

In contrast to childhood cancer care, adult models of cancer care are more patient- and disease-focused, in which the patient typically makes the decisions in consultation with the physician. Psychosocial support is not always part of standard care, thus being offered most often when problems have already arisen [12]. The staff/patient ratio is much lower compared to pediatric oncology, and care is dispersed among many departments and centers [12,126].

Cancer survivorship care for late effects in older adults is tumor-specific. Which long-term and late effects arise and how often depends on patient and cancer (treatment) characteristics [146]. Older adult cancer survivors have extensive guidelines available, just as pediatric survivors, through the National Comprehensive Cancer Network (NCCN) [147,148]. The guidelines for survivorship provide recommendations regarding screening, evaluation, and treatment, as well as preventive health (e.g., healthy lifestyles) and late and long-term effects (e.g., anxiety, lymphedema).

The Institute of Medicine (IOM) introduced survivorship care plans (SCPs) in 2006 as a possible solution to the lack of coordination of care between active treatment and long-term survivorship. According to the IOM, survivorship should be recognized as a distinct phase in cancer care in which survivors’ concerns should be addressed [118]. SCPs are aimed to inform, support communication, and facilitate self-care of the cancer survivor regarding their cancer diagnosis, treatment, and follow-up [149,150]. It provides an overview of the survivor’s diagnosis and treatment, including follow-up schedules, recommendations for early detection, and management of treatment-related effects and other health problems [118,151]. Despite widespread effort, the use of SCPs in practice remains suboptimal, and evidence regarding their impact on survivors’ outcomes is limited [150,151,152,153]. Hill and colleagues performed a systematic review and meta-analysis of care plan outcomes suggesting that SCPs seem feasible and can have a positive impact on HCPs’ knowledge (e.g., regarding late effects, survivorship care), but there is a lack of evidence that SCPs have an impact on the patient-reported outcomes of survivors [151]. However, whether this is due to SCP ineffectiveness or issues regarding implementation, for example, remains yet unclear. Nevertheless, the American College of Surgeons, for example, no longer requires SCPs to maintain institutional accreditation.

### 5.2. Challenges for AYA Survivorship Care

With regard to survivorship care, AYAs again fall in between the dichotomy of risk-based age-centered follow-up for childhood cancer survivors and disease-centered follow-up for adult cancer survivors, both with thorough survivorship guidelines. As a small sub-set of the overall adult cancer survivorship population, AYAs have unique needs relating to their age, developmental stage, and potential years of life lost to disability or sub-optimal health and well-being. AYA cancer survivors have many unaddressed concerns when they transition out of active cancer treatment, including the already described long-term and late effects from treatment, ongoing psychosocial issues, and navigating follow-up care [118,137]. Factors hindering the reception of survivorship care on a patient level include, among others, the patient’s poor knowledge of cancer history and risk of late effects, reluctance to return to the hospital, and logistical reasons [12,25]. When medical treatment is over, the support delivered during treatment may diminish as there will be less contact between the healthcare team and the patient. Some patients may not have access to specialized services, as they remain with their treating specialist to obtain their follow-up, focusing primarily on relapse detection [46]. In addition, many AYA survivorship issues are still poorly understood and possibly neglected [16,66,73,118,137,154]. Because both HCPs in hospitals and general practitioners (GPs) only see a limited number of AYAs in their practice, they are often unfamiliar with AYA-specific issues and have limited expertise in AYA specific care [12,46,118,136]. Being unfamiliar with this patient population may lead to the provision of suboptimal follow-up care. All things considered, at this moment in time it is questionable which survivorship model is best suited for use within the AYA cancer survivorship population and who should be responsible for this follow-up care.

### 5.3. Models of AYA Survivorship Care and Services Needed

A recent report from CanTeen Australia described the transition into survivorship when treatment is over as a complex process, which should ideally be supported by multiple services addressing the care needs of AYAs [77]. These services should focus on: (1) education about cancer (treatment) and related effects, (2) surveillance, screening, and treatment for medical, long-term, and late effects, (3) psychosocial support for reintegration, appropriate for the developmental phase of the AYA, (4) peer support (5) and having access to legal and/or financial support. They are currently integrating these elements in their AYA programs.

Nevertheless, maintaining the provision of care may be challenged by the wide variety of hospitals where AYAs are treated and the high mobility of this population [10]. However, as AYA survivors may have a long life ahead of them while potentially suffering from the biopsychosocial issues described earlier, it is of utmost importance to establish long-term AYA survivorship care for AYA survivors, regardless of where initial treatment took place. The NCCN developed clinical practice guidelines for AYA oncology addressing AYA specific issues and management considerations, recommended interventions, and education for HCPs [10,41]. In addition, different risk-based long-term survivorship models of care are available, which involve a screening, surveillance, and prevention plan based on the characteristics of the AYA survivor. Three AYA survivorship models of care highlighted by the NCCN include: (1) cancer center follow-up, led by an oncology team or a center of expertise, (2) primary care follow-up provided by the primary care provider, nurse practitioner or family practice physician, and (3) shared-care follow-up, led by a team of HCPs including primary care provider and oncologist. Which survivorship model is best to apply depends on several factors such as available resources [10,41].

### 5.4. Examples of AYA Survivorship Care

Fortunately, there are some great examples of AYA survivorship care. The Princess Margaret AYA program at the Princess Margaret Cancer Centre in Ontario, Canada, is one of the programs that integrated survivorship care as a core element [73,155]. Within their AYA program (those aged <39), concerns related to both cancer as well as ‘regular life’ are addressed by a clinical nurse specialist (CNS) who connects the AYA with the right services and resources needed. AYAs can meet the CNS either through self-referral or referral by an HCP: the latter can directly refer to services or to the AYA program. After gaining insight into the patient’s needs, the CNS provides teaching and resources to address these needs, also on a longitudinal basis when needed. Resources focus on but are not limited to financial support, fertility, exercise, nutrition, psychosocial support, sexual health, and advanced cancer. Both the comprehensive assessment as well as the survivorship treatment plan are tailored to the individual. The survivorship strategy includes an MDT, disease site-specific care plans, and the provision of both information and education by the CNS. Survivorship care is built on self-management principles and provides comprehensive, ongoing care across the age spectrum [73]. Recently, the dedicated AYA program was evaluated, after which the incremental added benefit of the program was declared in several key domains, including information provision regarding fertility, physical appearance, work, and school [156]. The essence of the program was about support, education, triage, and navigation. AYAs were satisfied with the offered supports, and even when they were very satisfied with their primary team, the program could still be of added value. Over time, more AYA consultations took place, showing an increased awareness and use of the program. This all may be the result of the program having identified key gaps in resources and care in AYA specific domains and having time invested in identifying AYA specialists, developing peer support networks, and creating resources addressing fertility issues, for example.

## 6. Future Directions

### 6.1. AYA Cancer Survivorship Research

It is key to (1) pool different types of data (patient-reported outcomes (PROs), clinical and biological data) across organizations and/or countries and (2) to create representative cohort studies, including all AYA ages and diagnoses, in order to make improvements in the AYA oncology field [3,10,46,157,158]. The results of these big data approaches, possibly linked to national registry databases, provide insight in subgroups of AYAs who are at risk of impaired outcomes and why, and may form the basis for further development and promotion of AYA cancer survivorship care, including widely available evidence-based AYA healthcare programs and guidelines. Eventually, it may evolve national policies to improve AYAs’ outcomes [77,157].

ESMO/SIOPE’s AYA Working Group and Salsman and colleagues both expressed several research barriers that need to be addressed first in order to compare and improve AYAs’ outcomes, including, amongst others, a lack of AYA-specific outcome measures [18,159]. Salsman and colleagues highlight, for example, the need for AYA-specific measurement tools that are valid, reliable, and cross the full AYA age spectrum in order to perform research and gain insight into the needs, issues, and health status of AYA cancer survivors [159]. Defining a standardized core outcome set with PROs and clinical outcomes, specifically for AYAs, may lead to the collection and report of AYA-specific standardized outcomes, which will enable the comparison of outcomes across different levels (e.g., patients, studies, countries). The input of AYAs themselves is of importance to compose core outcome sets and develop AYA-specific outcome measures that are relevant to them, take into account their developmental phase, and target their needs [137,160]. Table 2 provides a summarizing overview of the key points of AYA-specific survivorship research.

### 6.2. AYA Cancer Survivorship Care

Research findings can subsequently provide evidence-based input for improving AYA cancer survivorship care, including:

- Leading to improved information provision and communication among AYA cancer survivors and HCPs. Although the use of SCPs among cancer survivors in general was limited, they may serve their purpose among AYA cancer survivors, as Hydeman and colleagues highlight the desire of AYAs for more information regarding late and long-term biopsychosocial issues with which they are dealing [118]. Many AYA cancer survivors do not adhere to recommended tumor-specific survivorship care, which may be due to poor knowledge of the possible late effects, treatment, and health maintenance, underinsurance, costs, or reluctance to go back to the hospital [12,25,118]. AYAs should be empowered to assume ownership for their care and to have support for personal transitions (e.g., school, employment, and family planning) and practical issues (e.g., insurances and finances) [43]. As there is a lack of communication around AYA-specific issues, the SCP as part of standard care may facilitate key information provision [118]. An SCP may be beneficial for AYA cancer survivors to transition back to ‘normal life,’ e.g., by providing education and information regarding their treatments’ long-term and late effects and lifestyle recommendations [12,126]. It is, however, important to tailor SCPs to the developmental phase of AYAs [118,161].
- Gaining knowledge about specific (sub) groups at risk may lead to the development of risk-stratified AYA survivorship programs, guidelines, and surveillance strategies. Within pediatric oncology, survivorship care has been associated with improvements on both the patient level (e.g., improved patients’ knowledge regarding treatment and risks of late effects) as well as on a system level (e.g., fewer emergency room visits, hospitalizations, and better surveillance practices) [149]. The potential to mitigate the severity of late effects with early detection and management through survivorship care programs offers a great financial opportunity for healthcare systems [155]. A core outcome set, as described previously, can promote the development of quality care standards and guidelines and improves AYA-specific healthcare. Performance indicators, which can be part of an AYA-specific core outcome set, can improve quality of care but can also serve as a benchmark to achieve outcome-improvement goals and provide the information needed to make policy-related decisions and resources allocation. In Canada, besides an AYA-specific survivorship program, AYA-specific system performance indicators have been established by a national, multidisciplinary panel of AYA oncology experts [73,155]. Identifying system performance indicators specifically for AYAs may be applied internationally to monitor, evaluate, and benchmark the progress of (inter)national cancer care programs and institutions. Adequately resourced AYA care programs are, on a system level, more sustainable when ensured by a governmental policy [12,123,137], with a clear model of care with key performance indicators [124,126,162]. Table 2 provides a summarizing overview of the key points of AYA-specific survivorship care.

## 7. Conclusions

There are significant costs of being an AYA cancer survivor related to the individual and society. We are at the beginning of a journey to understand how to provide the best surveillance, and follow-up care that will enable HCPs and empowers AYA cancer survivors to (1) minimize the risk of adverse long-term and late effects and (2) mitigate the multiple factors that potentially negatively affect subsequent morbidity and mortality. Special attention should be paid to the unmet needs of AYAs living with an uncertain and poor prognosis [17]. Although lessons can be learned from the published evidence in pediatric and adult oncology survivors, not all are applicable to AYAs. We need to gain insight into which specific subgroups of AYAs are at risk of certain survivorship issues with the help of AYA specific research, improve the information provision and communication to create awareness among AYAs and HCPs, and empower them to act upon it. For this, international collaborative research efforts are needed, wherein diverse data are pooled, to inform the development of AYA cancer survivorship care programs, come to specific AYA cancer survivorship guidelines, and develop performance indicators to evaluate (future) care programs. All these efforts are needed to ensure the quality of survival for AYAs with cancer.

## Figures and Tables

**Table 1:** Age-standardized 5- and 10-year relative survival (RS) by primary cancer type in adolescents and young adults diagnosed between 1998 and 2016 (SEER 18, Regs Research Data, Nov 2018 submission) in rank order of 10-year RS.

Cancer Type	*n* at Risk	5-Year % RS (95%CI)	10-Year % RS (95%CI)
10-year survival > 80%			
Thyroid	43,504	99.8 (99.7–99.8)	99.7 (99.5–99.8)
Appendix (NET) ^a^	971	98.4 (96.9–99.2)	99.1 (97.1–99.7)
Lung and Bronchus (NET) ^b^	920	97.9 (96.7–98.6)	96.2 (94.7–97.3)
Testis	25,835	95.9 (95.7–96.1)	95.6 (95.3–95.8)
Melanoma of the Skin	29,489	95.4 (95.2–95.6)	93.8 (93.5–94.1)
Salivary Gland	1736	94.9 (93.9–95.7)	92.4 (91.2–93.5)
Hodgkin’s lymphoma ^c^	17,494	94.0 (93.6–94.2)	91.8 (91.4–92.2)
Ovary, non-epithelial ^d^	2250	92.5 (91.6–93.4)	91.1 (89.9–92.1)
Uterus ^e^	6318	89.5 (87.9–91.0)	87.5 (85.8–89.1)
Larynx	513	90.2 (87.4–92.5)	85.5 (80.6–89.2)
Vulva vaginal ^f^	666	83.4 (79.4–86.7)	80.5 (76.2–84.2)
Cervix Uteri	14,702	81.8 (80.6–83.0)	80.5 (79.2–81.7)
10-year survival 60–80%			
Pancreas (NET) ^g^	428	86.7 (82.7–89.8)	79.2 (73.3–83.9)
Kidney ^h^	7359	82.0 (80.8–83.2)	79.2 (77.8–80.6)
Non-Hodgkin Lymphoma ^i^	17,403	81.1 (80.6–81.6)	79.1 (78.5–79.6)
Tonsil **	492	82.0 (74.9–87.2)	78.5 (71.0–84.3)
Appendix (non-NET) ^j^	818	84.0 (81.7–86.1)	75.7 (72.2–78.8)
Small intestine	935	79.2 (76.2–81.9)	74.4 (71.2–77.3)
Breast	42,967	82.0 (80.8–83.1)	73.6 (72.3–74.9)
Bladder	759	75.2 (71.2–78.7)	71.5 (67.1–75.4)
Penis **	204	69.5 (59.9–77.2)	69.0 (59.3–76.8)
Ovary, epithelial ^k^	3544	74.4 (72.9–75.9)	68.0 (66.2–69.7)
Tongue	1439	69.0 (66.1–71.6)	67.5 (64.6–70.3)
Bones *	4061	72.3 (71.1–73.5)	67.3 (65.9–68.6)
Nasopharynx **	1229	74.1 (71.7–76.2)	66.2 (63.5–68.8)
Soft tissue sarcoma ^l^	6876	69.2 (68.3–70.1)	64.6 (63.6–65.5)
Prostate	473	64.1 (55.2–71.7)	63.8 (54.9–71.5)
Multiple Myeloma	1193	73.0 (68.5–76.9)	63.3 (58.4–67.7)
Rectum	4455	68.4 (66.5–70.3)	62.1 (60.0–64.2)
Leukemia ^m^ **	13,285	65.3 (64.6–66.0)	61.0 (60.3–61.7)
Anal ^n^	669	62.9 (55.1–69.8)	- *
10-year survival 40–60%			
Colon ^o^	4450	61.8 (60.0–63.5)	57.7 (55.8–59.5)
Brain	11,825	68.7 (68.0–69.3)	56.6 (55.7–57.4)
Sigmoid ^p^	2945	61.1 (58.4–63.7)	54.8 (51.9–57.6)
Rectosigmoid Junction	1368	60.9 (57.2–64.4)	54.1 (50.0–58.1)
10-year survival < 40%			
Pancreas (non-NET) ^q^	1369	38.8 (35.3–42.3)	32.1 (28.5–35.7)
Lung and Bronchus (non-NET) ^r^	3783	34.7 (32.6–36.8)	31.3 (29.1–33.6)
Stomach	3148	31.9 (29.9–34.0)	28.7 (26.6–30.8)
Liver	1346	33.8 (31.5–36.1)	27.3 (25.0–29.7)
Esophagus	519	23.2 (18.7–28.0)	21.2 (16.8–26.0)
Intrahepatic Bile Duct	236	19.5 (14.7–24.9)	- *

Abbreviations: NET = Neuroendocrine tumor, CI: confidence interval. * Could not be estimated due to low patient numbers in one or more age groups; ** Summary Stage 2000 (1998+) = blank; See Appendix B for additional recoding information.

**Table 2:** Key points of AYA-specific survivorship.

**Focus on AYA-Specific Survivorship Issues** Physical, e.g., o Second malignancies o Cardiovascular disease o Endocrine dysfunction o Neurocognitive deficits o Fertility o Sexual dysfunction o Body disfigurement o Physical condition Psychological, e.g., o Psychological distress o Posttraumatic stress o Fear of recurrence Social, e.g., o Education, employment, and financial challenges o Relationships
**AYA Cancer Survivorship Research:** - Access to appropriate trials - Pooling different data sources (e.g., PROs, clinical outcomes) - Development of standardized core outcome set
**AYA Cancer Survivorship Care:** - Improving information provision and communication (e.g., SCP) - Access to preventative measures (e.g., HPV vaccination) - Access to appropriate treatment and expert services - Development of AYA cancer survivorship programs (risk-stratified) - Development of evidence-based AYA cancer survivorship guidelines - Monitoring of provided care (e.g., performance indicators)

## Appendix A. Methods Used to Calculate the Age-Standardized 5- and 10-Year Relative Survival

SEER*Stat version 8.3.8 and STATA version 16.1 were used to collect and analyze the data. Relative survival estimates were determined with the –STRS– command in STATA and using the Ederer II method [163]. The United States Annual Life Tables: 1970–2017 were used for general population expected survival probabilities [164]. Outcomes were age-adjusted to the International Cancer Survival Standard 3 [165]. AYAs aged 15–39 years at their first malignant cancer diagnosis between 1998 and 2016 were included. Cancer types were classified based on the site recode International Classification of Diseases for Oncology-3 (ICD-O-3)/WHO 2008 (see Appendix B for exceptions), including local, regional, and distant staging based on the SEER Summary Stage 2000 (1998+). Patients with more than one cancer diagnosis were not included. Those with missing follow-up data were removed from the database (*n* = 175). Year of death, emigration, or the year 2016 determined end of follow-up, whichever came first.

## Appendix B. Tumor Type Categorization of Table 1 Explained, Based on Site Recode ICD-O-3/WHO 2008 and ICD-O-3 Histology/Behavior, Malignant

^a^ Includes: ICD-O-3: 8240, 8241, 8244, 8249.

^b^ Includes: ICD-O-3: 8240, 8241, 8249.

^c^ Includes: Hodgkin Extranodal and Nodal.

^d^ Includes: Sexcord stromal tumors (ICD-O-3: 8590, 8593, 8600, 8620, 8622, 8631, 8632, 8634, 8640, 8650, 8670), Sarcoma (ICD-O-3: 8800, 8803, 8805, 8806, 8810, 8851, 8854, 8890, 8896, 8900, 8901, 8910, 8920, 8931, 8933, 8935, 8950, 8951, 9044, 9120, 9260), Germ cell tumors (ICD-O-3: 8052, 8070, 8071, 8240, 8243, 9060, 9064, 9065, 9070, 9071, 9073, 9080-9085, 9090, 9091, 9100, 9101).

^e^ Includes: Corpus Uteri and Uterus, NOS.

^f^ Includes: Vulva and Vagina.

^g^ Includes: ICD-O-3: 8150-8153, 8155, 8156, 8240, 8249, 8452, 8680.

^h^ Includes: Kidney and Renal pelvis.

^i^ Includes: Non-Hodgkin Lymphoma Extranodal and Nodal.

^j^ Includes: ICD-O-3: 8000, 8010, 8140, 8210,8220, 8243, 8245, 8246, 8255, 8261, 8263, 8440, 8470, 8480, 8481, 8490, 8574, 8936, 9015.

^k^ Includes: ICD-O-3: 8010, 8012, 8013, 8020, 8021, 8033, 8041, 8045, 8046, 8050, 8120, 8140, 8144, 8230, 8246, 8255, 8290, 8310, 8313, 8320 8323, 8344, 8380-8382, 8401, 8410, 8441, 8442, 8450, 8460-8463, 8470-8472, 8480-8482, 8504, 8560, 8570, 8574, 8980, 9000, 9014, 9015.

^l^ Includes: Soft tissue including heart.

^m^ Includes Acute lymphocytic leukemia, Acute myeloid leukemia, Chronic lymphocytic leukemia, and Chronic myeloid leukemia.

^n^ Includes: Anus, Anal canal, and Anorectum.

^o^ Includes: Splenic flexure, Hepatic flexure, Ascending colon, Transverse colon, Descending colon, and Cecum.

^p^ Includes: Sigmoid colon.

^q^ Includes: ICD-O-3: 8000, 8001, 8010, 8012, 8013, 8020, 8021, 8032, 8035, 8041, 8046, 8050, 8070, 8140, 8154, 8160, 8230, 8246, 8255, 8260, 8290, 8440, 8450, 8470, 8471, 8480, 8481, 8490, 8500, 8504, 8550, 8551, 8560, 8574, 8890, 8936, 8963, 8971, 9120, 9260, 9364, 9473.

^r^ Includes: ICD-O-3: 8000, 8001, 8010, 8012, 8013, 8020-8022, 8031-8033, 8041, 8042, 8044–8046, 8050, 8051, 8070-8072, 8076, 8082, 8083, 8123, 8140, 8144, 8200, 8230, 8246, 8250–8255, 8260, 8310, 8323, 8333, 8345, 8370, 8430, 8471, 8480, 8481, 8490, 8507, 8550, 8560, 8574, 8576, 8580, 8680, 8711, 8800-8803, 8805, 8810, 8815, 8824, 8830, 8854, 8855, 8890, 8972, 8980, 9040, 9041, 9043, 9061, 9065, 9071, 9084, 9085, 9100, 9120, 9130, 9133, 9150, 9220, 9231, 9250, 9260, 9364, 9473, 9540, 9560, 9581.
